# Butylphthalide improves brain damage induced by renal ischemia-reperfusion injury rats through Nrf2/HO-1 and NOD2/MAPK/NF-κB pathways

**DOI:** 10.1080/0886022X.2023.2259234

**Published:** 2023-09-21

**Authors:** Jingjing Min, Qi Chen, Mengxiong Pan, Tan Liu, Qun Gu, Dongwei Zhang, Ru Sun

**Affiliations:** a Department of Neurology, The First People’s Hospital of Huzhou, First affiliated Hospital of Huzhou University, Huzhou, China; b Department of Nephrology, The First People’s Hospital of Huzhou, First affiliated Hospital of Huzhou University, Huzhou, China

**Keywords:** Renal I/R, brain injury, butylphthalide, blood-brain barrier, Nrf2/HO-1 pathway, NOD2/MAPK/NF-κB pathway

## Abstract

Renal ischemia-reperfusion (I/R) injury leads to irreversible brain damage with serious consequences. Activation of oxidative stress and release of inflammatory mediators are considered potential pathological mechanisms. Butylphthalide (NBP) has anti-inflammatory and antioxidant effects on I/R injuries. However, it is unclear whether NBP can effectively mitigate renal I/R secondary to brain injury as well as its mechanism, which are the aims of this study. Both renal I/R injury rats and oxygen and glucose deprivation cell models were established and pre-intervened NBP. The Morris water maze assay was used to detect behavior. Hippocampal histopathology and function were examined after renal I/R. Apoptosis and tube-forming capacity of brain microvascular endothelial cells (BMVECs) were tested. Immunohistochemistry and Western blot were used to measure protein expression of nuclear factor erythroid 2-related factor 2 (Nrf2)/Heme Oxygenase-1 (HO-1) pathway and NOD-like receptor C2 (NOD2)/Mitogen-activated protein kinases (MAPK)/Nuclear factor kappa-B (NF-κB) pathway. NBP treatment attenuated renal I/R-induced brain tissue damage and learning and memory dysfunction. NBP treatment inhibited apoptosis and promoted blood-brain barrier restoration and microangiogenesis. Also, it decreased oxidative stress levels and pro-inflammatory factor expression in renal I/R rats. Furthermore, NBP enhanced BMVECs’ viability and tube-forming capacity while inhibiting apoptosis and oxidative stress. Notably, the alleviating effects of NBP were attributed to Nrf2/HO-1 pathway activation and NOD2/MAPK/NF-κB inhibition. This study demonstrates that NBP maintains BBB function by activating the Nrf2/HO-1 pathway and inhibiting the NOD2/MAPK/NF-κB pathway to suppress inflammation and oxidative stress, thereby alleviating renal I/R-induced brain injury.

## Graphical Abstract

**Figure:**
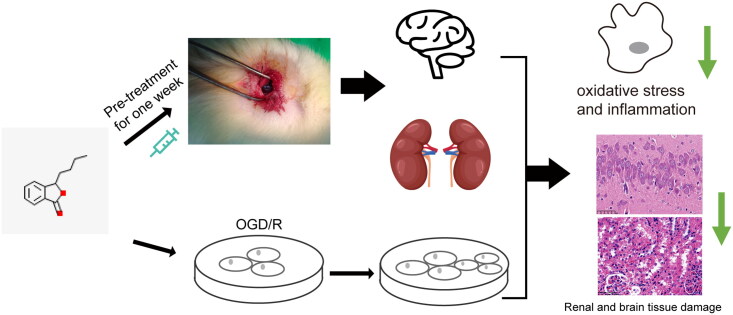

## Introduction

Renal ischemia-reperfusion (I/R) is a primary cause of acute kidney injury (AKI) in a variety of clinical settings, commonly in cases of hemorrhagic shock, partial nephrectomy, and renal transplantation [1]. Extensive research and epidemiological data indicate that the restoration of perfusion can induce damage to distal organs, leading to multiple organ dysfunction [2]. In particular, the brain is a highly perfused blood flow organ in the body and is very sensitive to I/R injury and the adverse effects of renal I/R injury on brain tissue have been widely reported [3,4]. Specifically, renal I/R not only causes apoptosis and inflammatory activation in brain tissue but also oxidative damage to the hippocampus [5,6]. Ultimately, it causes irreversible damage to neural cells, with serious consequences. However, there is a lack of definitive treatment for secondary renal I/R brain injury, so it is important to investigate its molecular mechanisms to explore active and effective prevention and treatment measures.

The mechanism of renal I/R injury is complex, involving a series of pathological processes such as inflammatory response, oxidative stress injury, apoptosis, and microcirculatory disorders [7]. Inflammatory responses play an important role in brain injury following AKI [8]. Under ischemic and hypoxic conditions, the massive release of inflammatory mediators disrupting the integrity of the blood-brain barrier (BBB) is the main cause of irreversible hippocampal damage [9]. Malek M et al. also found that the immune response and disturbance of BBB permeability following AKI triggered an inflammatory cascade and brain damage [10]. In addition, oxidative stress injury is likewise a key factor mediating brain dysfunction after renal I/R injury [11]. Additionally, a study demonstrated that AKI induces synaptic plasticity impairment and neurocognitive dysfunction through the modulation of oxidative stress in the hippocampus [12]. Evidence suggests that the nuclear factor erythroid 2-related factor 2 (Nrf2)/heme oxygenase-1 (HO-1) pathway was inhibited after renal I/R [13]. Nrf2 agonists have been found that it can improve renal I/R injury and its remote brain injury [14]. In addition, p38 mitogen-activated protein kinase (p38-MAPK) is associated with damage caused by reactive oxygen species and is significantly activated in the kidney after I/R [15]. Researchers have also demonstrated that the p38-MAPK/nuclear factor kappa-B (NF-κB) pathway was activated in the kidney after I/R [16]. It is clear that renal I/R-induced brain injury is the result of a combination of factors and that suppression of inflammation and oxidative stress levels is a promising therapeutic target.

Brain microvascular endothelial cells (BMVECs), a key component of BBB, with oxygen and glucose deprivation (OGD)/reoxygenation (OGD/R) treatment commonly used to study the I/R injury of BBB [17]. One study shows that hypoxia/reoxygenation destroys the Tight junction complex of human BMVECs [18]. The Nrf2/HO-1 pathway has been shown to be involved in antagonizing oxidative stress damage in ischemic stroke animals and H/R-treated endothelial cells [19]. One study reported that inhibiting MAPK may be involved in improving OGD/R injury to microvascular endothelial cells [20]. Additionally, suppression of p38-MAPK and NF-κB pathway can recover the inflammation level in the OGD/R-induced BMECs injury model [21]. Based on the above studies, regulation of the Nrf2/HO-1 and the p38-MAPK/NF-κB signaling pathway have an association with renal I/R-induced brain injury.

Current research suggests that pharmacological pretreatment may reduce organ damage by suppressing levels of inflammation and oxidative stress [22]. Among them, dl-3-n-butylphthalide (NBP) pretreatment has been found to have a significant protective effect on I/R injury [23]. NBP is a synthetic compound derived from l-3-n-butylphthalide, which is isolated from seeds of *Apium graveolens*. It was approved by the China Food and Drug Administration for the treatment of ischemic stroke in 2002 [24]. NBP attenuates myocardial I/R injury by regulating cardiac mitochondrial autophagy through inhibition of oxidative stress [23]. Sun et al. also found that NBP treatment attenuated I/R-induced histopathological changes while inhibiting inflammation and oxidative stress in mouse skeletal muscle [25]. Besides, due to its anti-inflammatory and antioxidant activities, NBP has been shown to have neuroprotective effects and is approved by the State Food and Drug Administration for the treatment of ischemic stroke [24,26]. However, the role of NBP in secondary renal I/R brain injury and molecular mechanisms are poorly studied. This study hypothesizes that NBP has a protective role in renal I/R injury and may inhibit secondary brain injury.

The project aim is to investigate the protective effect of NBP on the brain and its molecular mechanism by establishing a rat model of renal I/R injury and an OGD/R cell model. This provides new ideas for the prevention and treatment of secondary renal I/R brain injury and provides a theoretical basis for the clinical application of NBP.

## Materials and methods

### Animal ethology

Twenty-four Sprague Dawley (SD) rats weighing 200 g (6-week-old) were purchased from Shanghai Jihui Experimental Animal Breeding Co. SCXK (Shanghai) 2017-0012. All animal experiments were approved by the Animal Experimentation Ethics Committee of Zhejiang Eyong Pharmaceutical Research and Development Center (approval No. ZJEY-20211028-02).

### Establishment of renal I/R rat model

SD rats were randomly divided into four groups of six rats each: control group, sham group, I/R group, and NBP + I/R group. The noninvasive arterial clamping of the bilateral renal pedicle was used to prepare a rat renal I/R model according to the method that has been reported [27]. After the rats were anesthetized by 3.5% isoflurane and maintained with 1.5–2% isoflurane, an abdominal incision was made to expose and ligate the bilateral renal pedicle, and within 1 min of ligation the kidneys began to darken, turning purple-black after 45 min of ischemia. In other words, rats maintain renal ischemia for 45 min. Subsequently, they were performed reperfusion for 24 h. In the sham group, only the bilateral renal pedicle was exposed and sutured. The control group rats were not treated in any way. NBP (5 mg/kg/d) (CSPC, China) was intraperitoneally injected into the NBP + I/R group for one week continuously before the modeling [28]. The NBP was diluted to 5 mg/mL in saline. The NBP was provided by the pharmacy of the First People’s Hospital of Huzhou, China. Control, sham, and I/R groups were injected with the same volume of saline instead. After surgery, 100,000 units of penicillin sodium were intramuscularly injected once for anti-infection and rats in each group were given free access to water and food. The experimental operation was carried out by professional animal experimental technicians.

### Morris water maze assay

The Morris water maze assay was used to observe hippocampal lesioning [29]. Rat movements were tracked using a camera and Topscan software (Cleversys, Reston, VA) as in previous reports [30]. Briefly, the rat was consecutively trained for 5 d, where the localization navigation experiment lasted 4 d and the spatial search experiment for 1 d. During training trials, escape latency was recorded as the time taken for animals to reach the hidden platform. On the probe trial, the platform was removed and times of crossing the platform location and the duration of activity in the platform were recorded to indicate the degree of memory consolidation.

### Sample collection

After rats were anesthetized by inhalation, peripheral blood, brain tissue, and kidney tissue were collected. Rat hippocampal tissues were isolated according to the previously reported method [31]. Some of the tissues were fixed in 4% paraformaldehyde and used for subsequent histopathological experiments. The remaining brain tissues were preserved at −80 °C for molecular assays.

### Evaluation of inflammation, oxidative stress, and renal physiological function

Superoxide dismutase (SOD) and malondialdehyde (MDA) content in serum, hippocampal, and renal tissues were measured using SOD activity assay (Shanghai Biotec, S0103), lipid oxidation MDA assay (Biotec, S0131S) for evaluating anti-/oxidative stress. The expression of inflammatory factors interleukin (IL)-1β (Enzyme immunoassay, MM-0047R1), IL-18 (Enzyme immunoassay, MM-0194R1), tumor necrosis factor-α (TNF-α) (Enzyme immunoassay, MM-0180R1) in serum were measured using enzyme-linked immunosorbent assay (ELISA) kits. Additionally, serum was used to observe creatinine (Scr) and blood urea nitrogen (BUN) levels, the Rat Scr kit (Enzyme immunoassay, MM-0880R1) and BUN kit (Solarbio, BC1535) were used to assess renal function in rats and all procedures were carried out according to the kit instructions.

### Hematoxylin-Eosin (HE) staining

Paraffin sections of rat hippocampal and renal tissues were prepared. After treatment of the sections with xylene and gradient ethanol, HE staining (Sigma, H3136; E4009) was performed. The sections were adequately stained, dehydrated, transparently sealed, and microscopically examined. The semi-quantitative score of renal tissue is according to the Paller and coworkers report [32], which is generally based on renal tubular injury including tubular lumen obstruction, brush border loss, cytoplasmic vacuolization etc. Furthermore, evaluation of hippocampal tissue is conducted through necrotic neuron quantification in the CA1, CA3, and Dentate gyrus (DG) regions, as well as assessment of neural cell arrangement [14].

### Nissl staining

Sections of rat hippocampal tissue were dewaxed in xylene, soaked in gradient alcohol and washed with distilled water. 1% toluidine blue solution (OKA, 71041284) was used to stain the sections for 20–40 min and then washed off, the sections were dehydrated and transparent again in xylene. The Nissl microsomes were observed microscopically.

### Immunohistochemistry (IHC)

Paraffin sections of rat cerebral cortex were dewaxed in xylene and ethanol in turn, repaired with antigen, and then washed in 3% hydrogen peroxide solution. Primary antibodies: anti-Von Willebrand Factor (vWF) (ab287962, 1:50), anti- B-cell lymphoma-2 (Bcl-2) (ab32124, 1:250), anti-BCL2-Associated X (Bax) (ab32503, 1:250), anti-Caspase-3 (ab32351, 1:25) were added separately and incubated overnight at 4 °C. HRP-labeled secondary antibodies (CST, 7074) were added and microscopically examined. The IHC was analyzed using ImageJ (v2.0, National Institute of Health). Average Optical Density (AOD) = integrated optical density (IOD)/area

### Tunel staining

After repair of the rat cerebral cortical tissue sections by Proteinase K (Beyotime, ST532), 0.1% Triton was added dropwise to disrupt the cell membrane. After incubation in the buffer for 10 min at room temperature, the DAPI stain (abcam, ab104139) was used to re-stain the nuclei of the cells after the reagents had been added according to the instructions of the Tunel kit (Beyotime, C1090). The kit was fluorescently labeled with Cyanine 3 and the nuclei of apoptotic cells were stained red. The slices of each mouse were observed under a fluorescence microscope, and three fields of view (×200) were randomly selected to take photos. The number of TUNEL positive cells and the total number of DAPI labeled cells were counted using ImageJ, and the positive cell rate = number of TUNEL positive cells/number of DAPI labeled cells.

### Immunofluorescence

Immunofluorescence staining was used to detect the expression levels of claudin-5 (Affinity, AF5216) and ZO-1 (Proteintech, 21773-1-AP) in coronal sections of each group of rat brains [33]. Paraffin sections were initially dewaxed and hydrated and then placed in boiling antigen repair solution for 15 min. Sequentially, 5% Goat serum, the primary antibodies, and Goat Anti-Rabbit IgG H&L were added. It was observed under the microscope after the antigen-antibody reaction. The average fluorescence intensity was analyzed using ImageJ. Mean gray value (average fluorescence intensity) = Integrated Density/Area.

### Cell culture

Brain microvascular endothelial cells (BMVECs) were purchased from iCell Bioscience Inc (Shanghai, RAT-iCell-n001). Cells were cultured in DMEM high sugar medium (Gibco, REF12800-01710X1L) containing 10% FBS (Season, 11011-8615) and placed in a 37 °C, 5% CO_2_ incubator [34].

### OGD model establishment and cell grouping

OGD treatment was used to simulate an *in vitro* model of I/R. First, to determine the optimal NBP intervention concentration, BMVECs were randomly divided into a control group, an OGD model group, and different NBP intervention concentrations (0.1, 1, 10, and 100 μmol/L). The control group was cultured routinely, while the remaining groups were treated with OGD according to the reported method: the BMVECs cell medium was replaced with a sugar-free medium and incubated in an anaerobic incubator for 4 h. After restoration of oxygen and glucose, the incubation was continued for 24 h [35]. The administered groups were treated with different concentrations of NBP for 24 h prior to the OGD. MTT was used to assay cell viability in each group, and the NBP concentration at which cell survival was highest was used as the subsequent NBP intervention concentration. Cells were randomly divided into control, OGD, NBP + OGD, and NBP + MDP (Muramyl dipeptide) + OGD groups. In the NBP + MDP group, 10 μg/mL MDP (Abcam, ab287084), an NOD2 agonist, was added to incubate the cells for 24 h while NBP was treated [36,37].

### MTT

BMVECs were inoculated in 96-well plates and 10 μL of MTT solution (Beyotime, ST316) was added to each well after modeling according to the above grouping. After sufficient incubation, the OD value at 490 nm was measured and the survival rate of each group of cells was calculated.

### Cell tube formation assay

Cell tube formation assay was performed to assess cell angiogenesis ability [38]. Briefly, pre-chilled 96-well plates were incubated with 50 µL matrigel (Univ, 356234) per well for 45 min at 37 °C. 50 µL cell resuspension was added to each well at a concentration of 3.5 × 10^4^ cells per well. 4 h incubation at 37 °C was followed by photography and ImageJ software was used to analyze the number of lumen-like structures.

### Flow cytometry

Cells were collected after 24 h of treatment as described above, and the cell concentration was adjusted to 1 × 10^6^/mL. 500 μL of binding buffer (BD Pharmingen, 51-66121E) was added, the supernatant was discarded by centrifugation, 100 μL of binding buffer was added and mixed, then 5 μL of Annexin V-FITC (BD Pharmingen, 51-65874X) and 10 μL of PI (BD Pharmingen, 51-66211E) were added and mixed thoroughly; the reaction was performed for 15 min at room temperature and protected from light. 400 μL of binding buffer was added and the cells were detected by flow cytometry within 1 h. Apoptosis rate was measured by flow cytometry within 1 h.

### Western blot

Organizational and cellular proteins were extracted from rat cerebral cortex and whole BMVEC cells using RIPA Lysis Buffer (Beyotime, P0013C) in an ice bath, and the supernatant after centrifugation (14,000 × g, 5 min, 4 °C) was used for subsequent experiments. Cellular concentrations were determined by the BCA method (Solarbio, pc0020). The target bands were sequentially obtained by SDS-PAGE electrophoresis, membrane transfer, and antigen-antibody reaction, and finally developed by ECL chemiluminescence (Clinx, 610020-9Q). Information on the antibodies involved in the experiments is shown in Table 1. The gray value of protein bands was analyzed using ImageJ.

**Table 1.** The antibody Information of Western blot.

Antibody	Manufacturer	Cat. No	Dilution ratio
Caspase3 Antibody	Abcam	ab13847	1:500
Bcl-2 Antibody	Affinity	AF6139	1:1000
Bax Antibody	Affinity	AF0120	1:1000
claudin-5 Antibody	Affinity	AF5216	1:1000
ZO-1 Antibody	Proteintech	21773-1-AP	1:1000
Nrf2 Antibody	Proteintech	16396-1-AP	1:1000
HO-1 Antibody	Proteintech	27282-1-AP	1:1000
NLRP3 Antibody	Affinity	DF7438	1:1000
Caspase-1 Antibody	Proteintech	22915-1-AP	1:1000
VEGF Antibody	Proteintech	19003-1-AP	1:1000
VEGFR2 Antibody	Affinity	AF6281	1:1000
NOD2 Antibody	Affinity	DF12125	1:1000
p-ERK1/2 Antibody	Cell Signaling Technology	4370T	1:1000
ERK1/2 Antibody	Cell Signaling Technology	4695T	1:1000
p-JNK Antibody	Affinity	AF3318	1:1000
JNK Antibody	Affinity	AF6318	1:1000
p-p38 Antibody	Affinity	AF4001	1:1000
p38 Antibody	Affinity	AF6456	1:1000
p-P65 Antibody	Affinity	AF2006	1:1000
P65 Antibody	Affinity	AF5006	1:1000
p-IkBα Antibody	Affinity	AF5002	1:1000
IkBα Antibody	Affinity	AF2002	1:1000
β-actin Antibody	Affinity	AF7018	1:10000
Anti-rabbit IgG, HRP-linked Antibody	Cell Signaling Technology	7074	1:6000

### Statistical analysis

If measurement data between multiple groups meet normal distribution and homogeneity-of-variance test, one-way-ANOVA followed the Tukey test is used. For a normal distribution but uneven variance, the Dunnett’s T3 test or independent sample *t*-test was used. If not meet normal distribution, Kruskal-Wallis *H* test is used. All results were analyzed using SPSS 16.0 software (IBM, USA) with a significance level set at *p*-value < 0.05 and are presented as mean ± SD. All replicates (n) are biological replicates.

## Results

### NBP promotes recovery of learning memory capacity and suppresses levels of inflammation in rats with renal I/R

**Figure 1.**
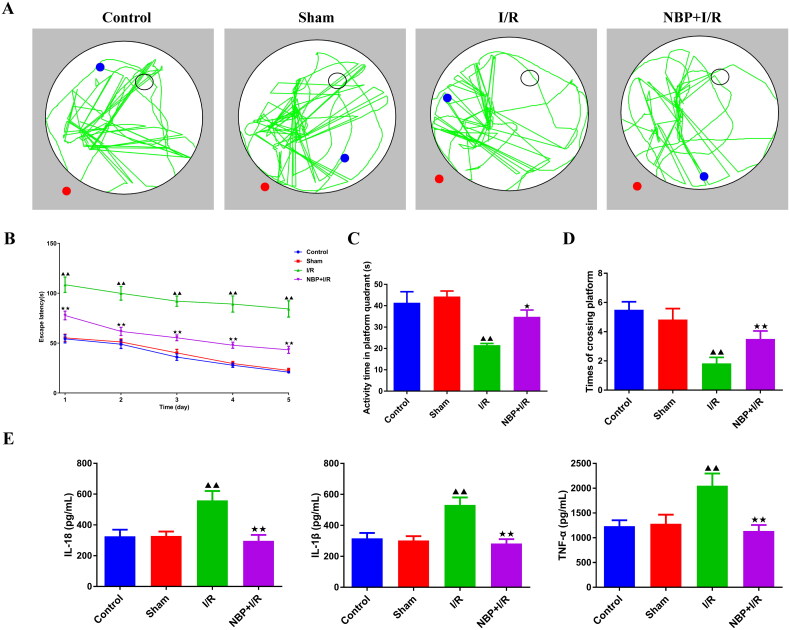
NBP promotes recovery of learning memory capacity and suppresses levels of inflammation in rats with renal I/R. (A-D) the Morris water maze Behavioral assay was used to examine the learning memory capacity of rats (*n* = 6). rat swimming path (a), rat escape latency (B), activity time in platform quadrant (C), and times of crossing platform (D) were shown (*n* = 6). (E) ELISA was used to measure the levels of IL-18, IL-1β and TNF-α in serum (*n* = 6). Data were presented as the mean ± standard deviation, ^▲^*p* < 0.05, ^▲▲^*p* < 0.01.vs. sham group; ^↔^*p* < 0.05, ^↔↔^*p* < 0.01.vs. I/R group. NBP: Butylphthalide; I/R: Ischemia/reperfusion; IL-18: Interleukin 18; IL-1β: Interleukin 1β; TNF-α: Tumor necrosis factor-α.

The Morris water maze assay was used to examine the learning memory capacity of rats (Figure 1A–D). The I/R group had longer escaped latency and less platform activity time and number of platform crossings than the sham group (Figure 1A–D). NBP pretreatment shortens the escape latency and increases the platform activity time and the number of platform crossings (Figure 1A–D). ELISA results indicated that inflammatory indicators IL-18, IL-1β and TNF-α in the serum were higher in the I/R group than sham group (Figure 1E). In contrast, the expression of inflammatory factors in the NBP pretreatment group was lower than that in the I/R group (Figure 1E).

### NBP alleviates kidney injury and reduces oxidative stress in rats with renal I/R

**Figure 2.**
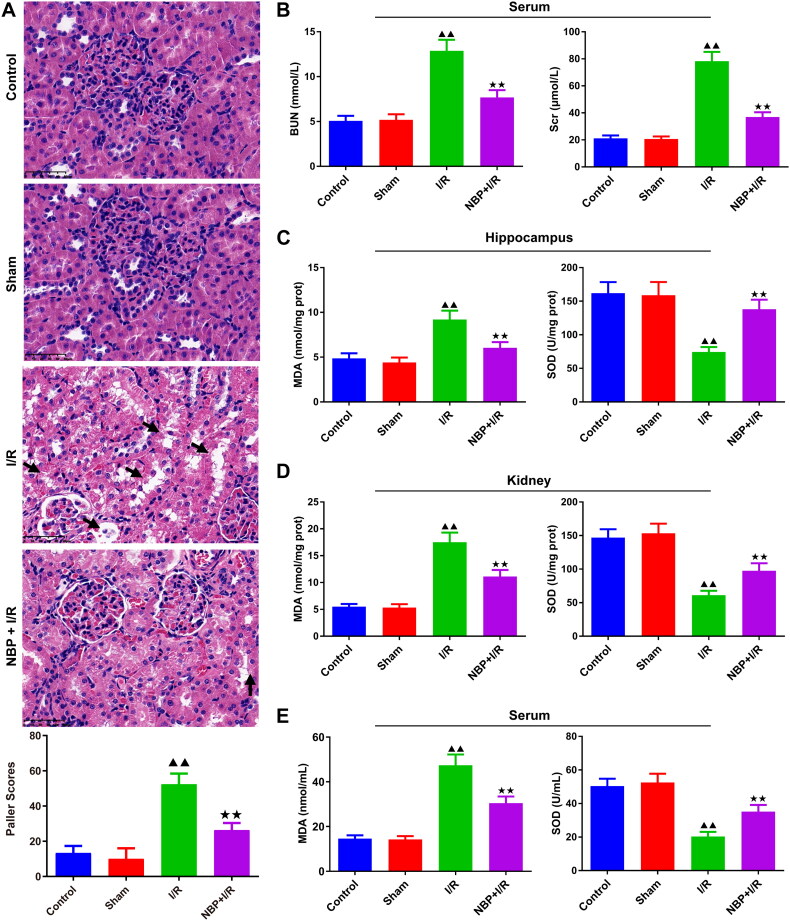
NBP alleviates kidney injury and reduces oxidative stress in rats with renal I/R. (A) HE staining was used to observe the histopathological changes in the rat kidney (magnification, ×400) (50 μm). (B) Serum levels of BUN and Scr were examined by kit. The expression levels of MDA and SOD in hippocampal tissue (C), renal tissue (D) and serum (E) (*n* = 6). Data were presented as the mean ± standard deviation, ^▲^*p* < 0.05, ^▲▲^*p* < 0.01.vs. sham group; ^↔^*p* < 0.05, ^↔↔^*p* < 0.01.vs. I/R group. HE: Hematoxylin-Eosin; BUN: Blood urea nitrogen; Scr: Serum creatinine; MDA: Malondialdehyde; SOD: Superoxide dismutase.

The kidney structure appeared normal and clear in both the control and sham groups, whereas significant renal tubular damage was observed in the I/R group (Figure 2A). In contrast, the renal tubules of the NBP pretreated group appeared to be intact, Additionally, the Paller score of the NBP pretreated group was significantly reduced (Figure 2A). The levels of BUN and Scr in serum were higher in the I/R group compared to the sham group, and NBP pretreatment reversed this change (Figure 2B). In addition, this study detected the SOD and MDA content in serum, hippocampal, and renal tissues, and the levels of inflammatory factors, creatinine, and BUN in serum (Figure 2C–E). The results showed that MDA was significantly elevated in the I/R group compared to the sham group, while SOD levels were reduced. Again, NBP pretreatment antagonized these changes (Figure 2C–E).

### NBP reduces hippocampal injury in rats with renal I/R

**Figure 3.**
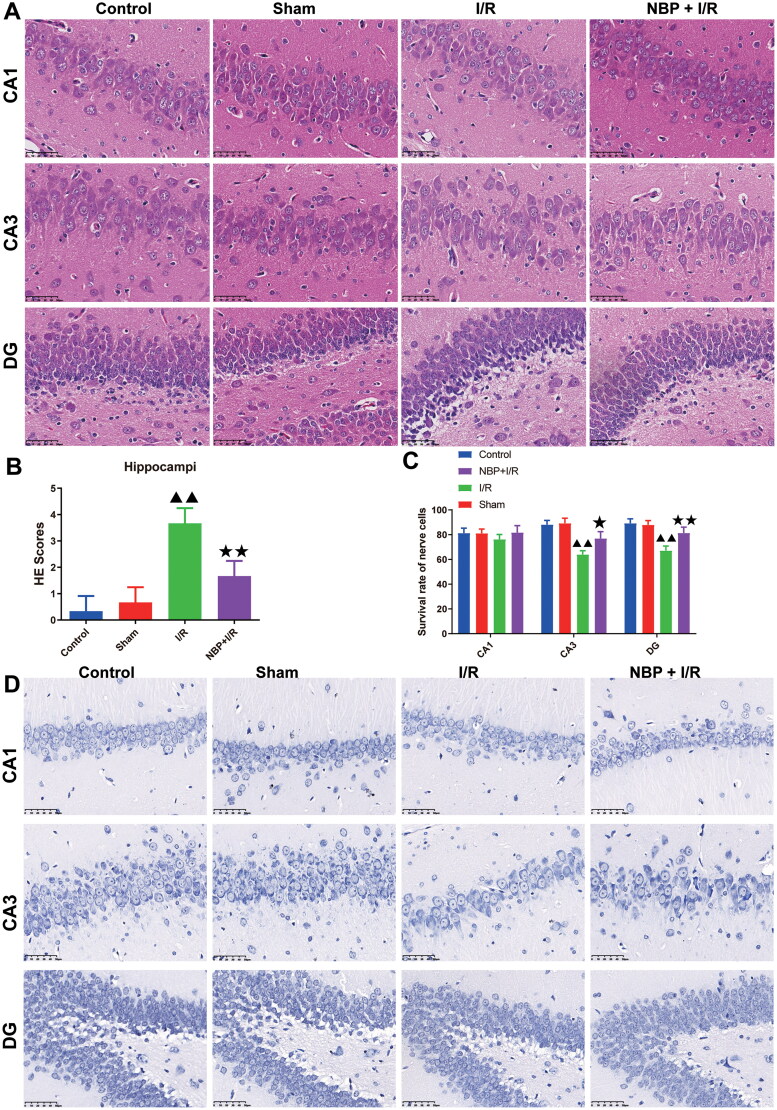
NBP reduces hippocampal injury in rats with renal I/R. (A, B) The histopathological Images and scores of the rat brain were observed by HE staining (magnification, ×400) (50 μm). (C, D) Nissl staining was used to detect changes in the number of neurons in the CA1, CA3 and DG regions of the hippocampus (magnification, ×400) (50 μm) (*n* = 3). Data were presented as the mean ± standard deviation, ^▲^*p* < 0.05, ^▲▲^*p* < 0.01.vs. sham group; ^↔^*p* < 0.05, ^↔↔^*p* < 0.01.vs. I/R group. DG: Dentate gyrus.

HE staining of hippocampus tissues revealed that the CA1, CA3, and DG regions of control and sham groups demonstrated normal brain tissue architecture (Figure 3A). Conversely, in the CA3 and DG regions of the I/R animal group, there was observed disordered neural cell organization and a significant decrease in neuronal count. However, these adverse effects were notably mitigated by NBP pretreatment (Figure 3A). The Histopathological score also corroborated the aforementioned findings (Figure 3B). In Nissl staining, the neuronal cells in the control and sham groups were light blue and cytoarchitecturally intact, whereas some neuronal cells in the CA3 and DG regions of the I/R group were vacuolated and less numerous; Compared with the I/R group, the NBP pretreatment group showed an increased number of cells and a more intact cell structure, suggesting a significantly higher neuronal survival rate in the rats (Figure 3C, D).

### NBP reduces the level of apoptosis and microvessel density in the cerebral cortex of rats with renal I/R

**Figure 4.**
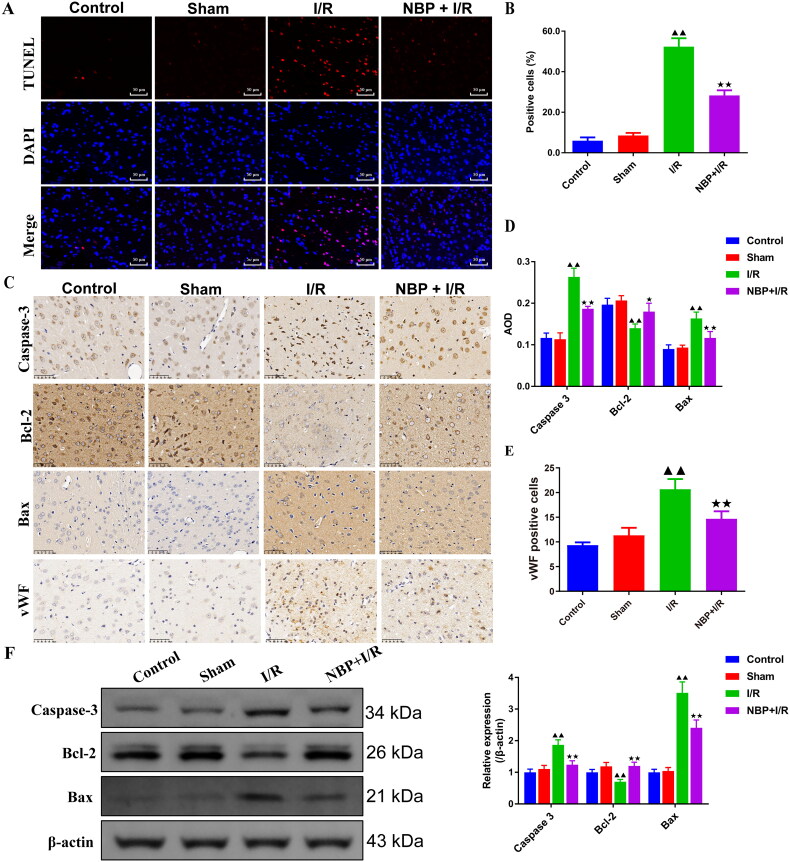
NBP reduces the level of apoptosis and microvessel density in the cerebral cortex of rats with renal I/R. (A-B) Tunel staining was performed to detect the effect of NBP pretreatment on the level of apoptosis in the cerebral cortex of rats with IR (magnification, ×200) (*n* = 3). (C-E) the expression levels of Caspase-3, Bcl-2, Bax and vWF in the brain tissues were determined by Immunohistochemistry (magnification, ×400) (50 μm) (*n* = 3). (F) Meanwhile, Western blot was used to detect the expression levels of Caspase-3, Bcl-2 and Bax in the brain tissues (*n* = 3). Data were presented as the mean ± standard deviation, ^▲^*p* < 0.05, ^▲▲^*p* < 0.01.vs. sham group; ^↔↔^*p* < 0.05, ^↔↔^*p* < 0.01.vs. I/R group. Bcl-2: B-cell lymphoma-2; Bax: Bcl-2 assaciated X protein; vWF: Von Willebrand Factor.

Tunel staining was performed to detect the effect of NBP pretreatment on the level of apoptosis in the cerebral cortex of rats with IR (Figure 4A–B). The apoptosis rate in brain tissue was significantly higher in the I/R group compared to the sham group; while it was lowered in the NBP pretreatment group compared to the I/R group (Figure 4A–B). Immunohistochemical and Western blot results suggested that the protein expression of Bax and caspase-3 was lower and Bcl-2 protein expression was higher in the NBP pretreated group compared to the I/R group (Figure 4C, D, F, and supplementary figure 1). Meanwhile, vWF protein in brain tissue was highly increased in the I/R group than the sham group, while vWF protein expression was reduced in the NBP pretreatment group (Figure 4C, E).

### NBP promotes BBB repair and neovascularization in renal I/R rats

**Figure 5.**
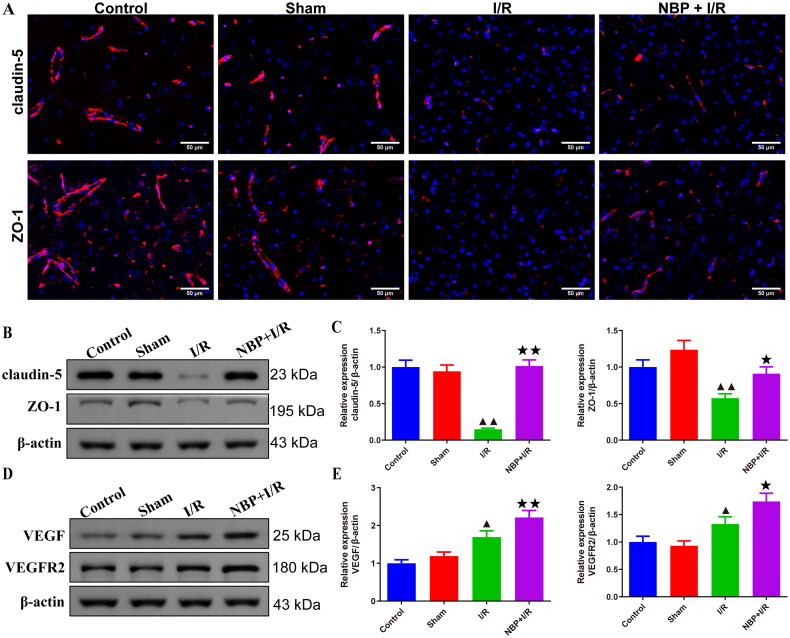
NBP promotes blood-brain barrier repair and neovascularization in renal IR rats. (A) Immunofluorescence staining was used to examine the effect of NBP pretreatment on the expression levels of claudin-5 and ZO-1 of the brain of I/R rats (magnification, ×200). the expression levels of Claudin-5, ZO-1 (B, C) and VEGF, VEGFR2 (D, E) in the rat brain were measured by Western blot (*n* = 3). Data were presented as the mean ± standard deviation, ^▲^*p* < 0.05, ^▲▲^*p* < 0.01.vs. sham group; ^↔^*p* < 0.05, ^↔↔^*p* < 0.01.vs. I/R group. ZO-1: Zonula occluden-1; VEGF: Vascular endothelial growth factor; VEGFR2: VEGF receptor-2.

Immunofluorescence results showed that the expression levels of both claudin-5 and ZO-1 in the cerebral cortex of the brain were reduced in the brains of rats in the I/R group compared to the sham group (Figure 5A). In addition, the results showed that the expression levels of claudin-5, ZO-1, VEGF and VEGFR2 were higher in the NBP pretreated rat brain compared to the I/R group (Figure 5B–E and Supplementary Figures 2 and 3).

### NBP activates Nrf2/HO-1 and inhibits NOD2/MAPK/NF-κB signaling pathway in brain tissue of renal I/R rats

**Figure 6.**
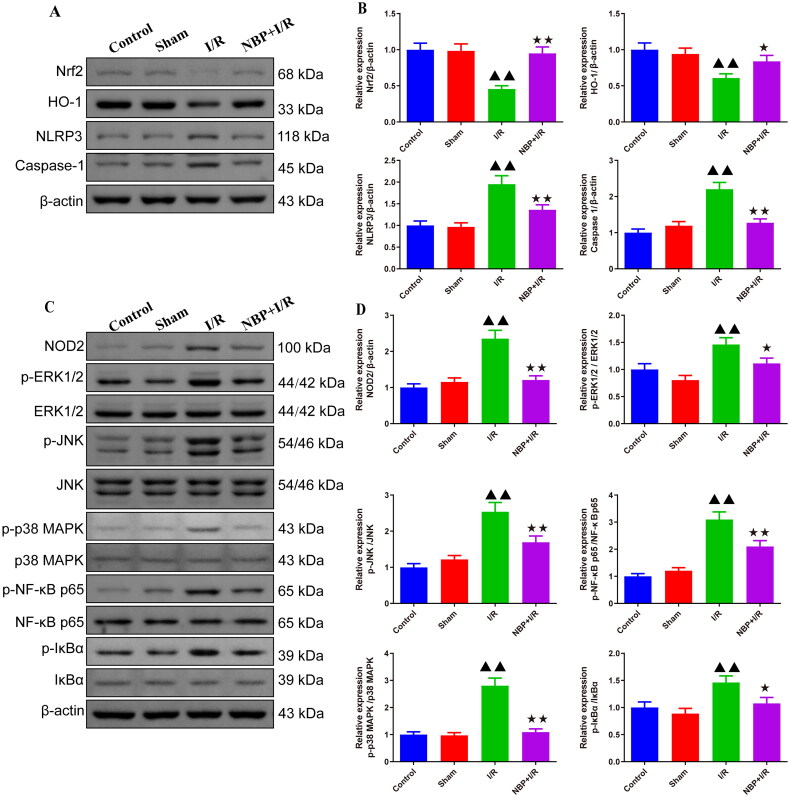
NBP activates Nrf2/HO-1 and inhibits NOD2/MAPK/NF-κB signaling pathway in the brain tissue of renal IR rats. (A-D) Western blot was used to detect the protein expression levels of Nrf2, HO-1, NLRP3, Caspase-1, NOD2, p-ERK1/2, p-JNK, p-p38, p-P65 and p-IkBa in the rat brain (*n* = 3). Data were presented as the mean ± standard deviation, ^▲^*p* < 0.05, ^▲▲^*p* < 0.01.vs. sham group; ^↔^*p* < 0.05, ^↔↔^*p* < 0.01.vs. I/R group. Nrf2: NF-E2-related factor 2; HO-1: Heme oxygenase-1.

Western blot results suggested that Nrf2 and HO-1 protein expression levels were decreased, while NLRP3, Caspase-1, NOD2, p-ERK1/2, p-JNK, p-p38 MAPK, p-NF-κB p65 and p-IkBa protein expression levels were increased in the brains of rats with renal I/R compared with the sham rats (Figure 6A–D, and Supplementary Figures 4 and 5). Compared with the I/R group, Nrf2 and HO-1 protein expression levels were increased and NLRP3, Caspase-1, NOD2, p-ERK1/2, p-JNK, p-p38, p-P65, p-IkBa protein expression levels were decreased in the brains of renal I/R rats with NBP pretreatment (Figure 6A–D and Supplementary Figure 4 and 5).

### NBP enhances cell viability and inhibits apoptosis by activating Nrf2/HO-1 and inhibiting the NOD2/MAPK/NF-κB signaling pathway in BMVECs

**Figure 7.**
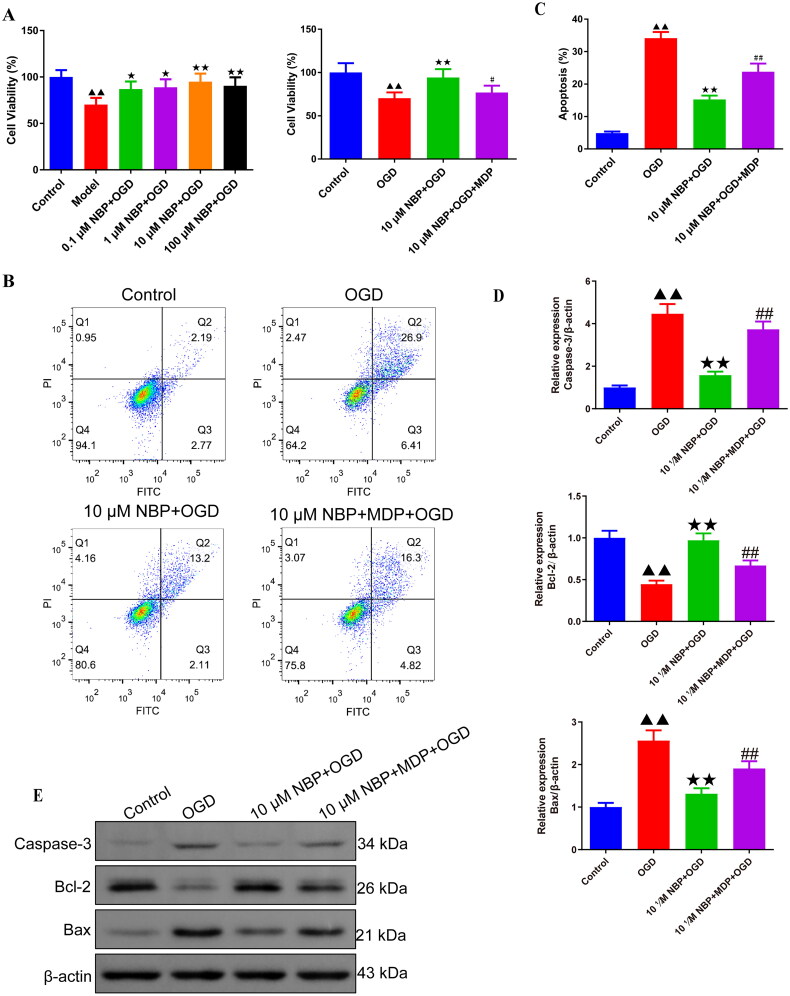
NBP enhances OGD-BMVECs viability and inhibits apoptosis. (A) MTT assay was carried out to detect the effect of NBP on the cell viability of BMVECs (*n* = 5). (B, C) Flow cytometry was used to examine the effect of different concentrations of NBP on OGD-BMVECs apoptosis (*n* = 3). (D, E) the expression levels of Caspase-3, Bcl-2, and Bax in the BMVECs were determined by Western blot (*n* = 3). ^▲^*p* < 0.05, ^▲▲^*p* < 0.01.vs. OGD group; ^↔^*p* < 0.05, ^↔↔^*p* < 0.01.vs. 10 μM NBP + OGD group; ^#^*p* < 0.05, ^##^*p* < 0.01.vs. 10 μM NBP + MDP + OGD group. OGD: Oxygen and glucose deprivation; BMVECs: Brain microvascular endothelial cells; MDP: Muramyl dipeptide.

**Figure 8.**
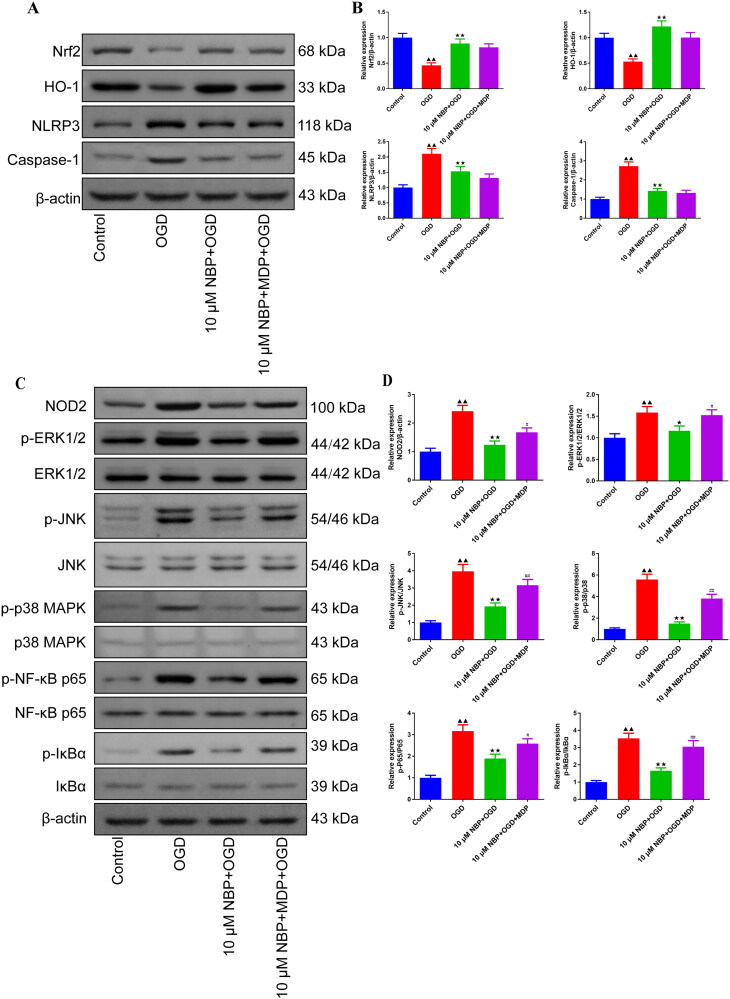
NBP Protects BMVECs by activating the Nrf2/HO-1 and inhibiting NOD2/MAPK/NF-κB signaling pathway. (A-D) Western blot was used to detect the protein expression levels of Nrf2, HO-1, NLRP3, Caspase-1, NOD2, p-ERK1/2, p-JNK, p-p38, p-P65 and p-IkBα in the total BMVEC protein (*n* = 3). ^▲^*p* < 0.05, ^▲▲^*p* < 0.01.vs. OGD group; ^↔^*p* < 0.05, ^↔↔^*p* < 0.01.vs. 10 μM NBP + OGD group; ^#^*p* < 0.05, ^##^*p* < 0.01.vs. 10 μM NBP + MDP + OGD group.

OGD/R cell model was used to explore the mechanism of NBP regulating NOD2 targets, inhibiting oxidative stress and inflammation of vascular endothelial cells to prevent brain tissue damage after renal I/R. Viability of BMVECs was reduced in the OGD group compared with the control group (Figure 7A). At the same time, NBP intervention increased cell viability compared to the OGD group and had the strongest effect at a concentration of 10 μmol/L (Figure 7A). However, MDP was able to reduce the promotion effect of NBP on cell viability (Figure 7A). MDP is Muramyl dipeptide, a NOD2 agonist [37]. Also, flow cytometry and Western blot results indicated that the inhibition of apoptosis in OGD cells by NBP was also blocked by MDP (Figure 7B–E, and supplementary figure 6). In addition, this study found higher levels of NOD2, p-ERK1/2, p-JNK, p-p38, p-P65, and p-IkBα protein expression of the 10 μM NBP + MDP + OGD group than the 10 μM NBP + OGD group (Figure 8A–8D and Supplementary Figures 7 and 8). The experimental results suggest that NBP may activate the Nrf2/HO-1 pathway and inhibit the NOD2/MAPK/NF-κB pathway to improve cell viability and antagonizes apoptosis.

### NBP promotes tube-forming capacity and suppresses oxidative stress and inflammation levels of BMVECs by activating Nrf2/HO-1 and inhibiting the NOD2/MAPK/NF-κB signaling pathway

**Figure 9.**
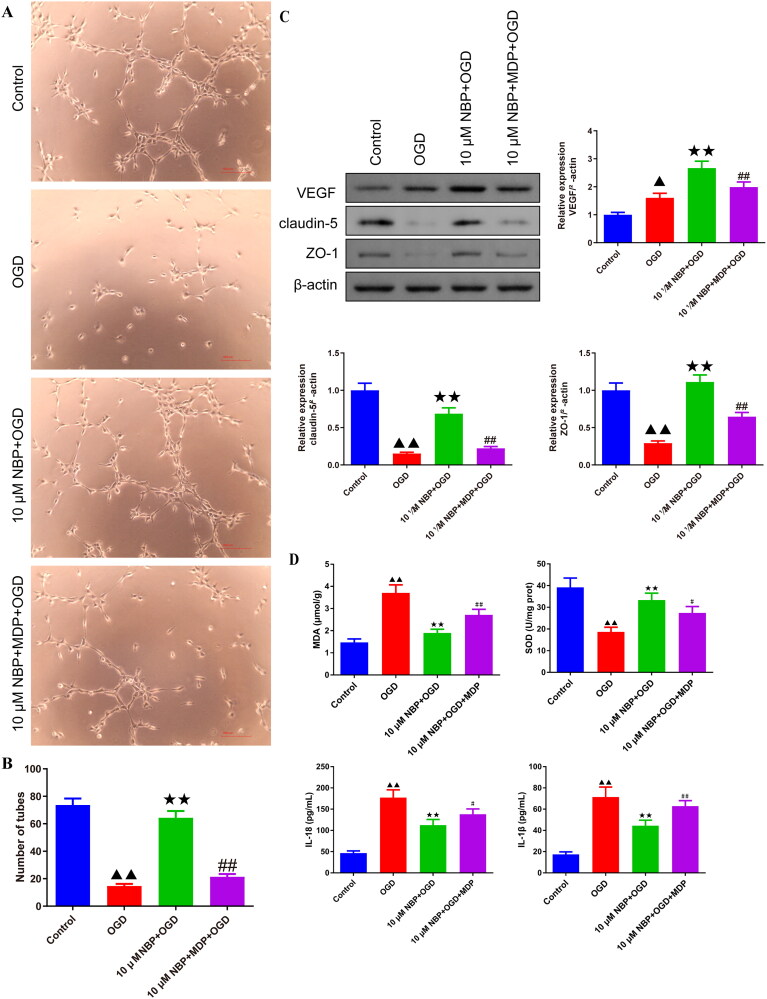
NBP promotes tube-forming capacity and suppresses oxidative stress and inflammation levels in the BMVECs. (A, B) Effect of NBP on BMVECs tube-forming capacity was assessed and the number of tubes was counted (*n* = 3). (C) Western blot was used to examine the effect of NBP pretreatment on the expression levels of VEGF, claudin-5 and ZO-1 in OGD-BMVECs (*n* = 3). (D) In addition, the effect of NBP on the MDA, SOD, IL-18 and IL-1β in the supernatant of BMVECs was examined (*n* = 6). ^▲^*p* < 0.05, ^▲▲^*p* < 0.01.vs. OGD group; ^↔^*p* < 0.05, ^↔↔^*p* < 0.01.vs. 10 μM NBP + OGD group; ^#^*p* < 0.05, ^##^*p* < 0.01.vs. 10 μM NBP + MDP + OGD group.

Next, the effect of NBP on cellular tube-forming capacity was assessed (Figure 9A–B). The results showed that the number of tube-forming cells in BMVECs in the 10 μM NBP + OGD group was increased compared to the OGD group, while MDP antagonized this change (Figure 9A–B). The expression of claudin-5 and ZO-1 and angiogenic factor VEGF was higher in the BMVECs of the 10 μM NBP + OGD group compared to the OGD group, and MDP intervention reduced this alteration (Figure 9C and Supplementary Figure 9). Supernatant MDA, SOD, IL-18, and TNF-α levels were also detected in rat BMVECs cells (Figure 9D). Compared with the OGD group, the levels of MDA, IL-18, and TNF-α contents in rat BMVECs in the 10 μM NBP + OGD group were lower, and the levels of SOD contents were higher, while MDP addition partly offset the effect of NBP on these oxidative stress and inflammatory indicators (Figure 9D).

## Discussion

This study has confirmed that NBP pretreatment reduced renal I/R-induced secondary brain injury in rats. This study suggests that NBP exerts neuroprotection by attenuating levels of oxidative stress and inflammation, preventing apoptosis in BMVECs, and promoting the restoration of BBB function. This study has further examined the role of the NOD2/MAPK/NF-κB signaling pathway using the NOD2 agonists and has found that these effects may be mediated through NOD2/MAPK/NF-κB pathway inhibition, while simultaneously activating the Nrf2/HO-1 pathway.

Distal organ damage caused by renal I/R injury has been reported [2,3]. Here, this study also found that in addition to severe structural and functional impairment of renal tissue, renal I/R rats also resulted in learning and memory dysfunction in rats. Interestingly, this study found that NBP could partially restore learning and memory functions in renal I/R rats. However, the mechanism by which NBP acts is not clear. Studies have shown that disruption of BBB integrity is secondary to irreversible hippocampal damage and is a major cause of learning and memory deficits [9]. Also, increased BBB permeability triggers neurological dysfunction in rats [39]. Therefore, this study hypothesized that NBP pretreatment alleviates learning and memory deficits in renal I/R rats in relation to blood-brain barrier protection. The BBB is mainly composed of BMVECs and astrocytes [40], so BMVECs were used to explore potential biological mechanisms of NBP.

Oxidative stress and the release of inflammatory mediators are the main pathological mechanisms of renal I/R injury and the main cause of BBB destruction [41–44]. NBP can protect endothelial cells from injury by ameliorating inflammatory and oxidative stress responses [45]. In this study, high expression of MDA, IL-18, IL-1β, TNF-α and reduced SOD were antagonized by NBP pretreatment on renal I/R and OGD-BMVECs models suggesting NBP protected BBB from damage through anti-oxidative stress and inflammatory response. The Nrf2/HO-1 pathway has been focused as a major regulatory target for intracellular defense against oxidative stress [46]. Meanwhile, NBP pretreatment inhibited oxidative stress levels in microglia, retinal cells, and cardiomyocytes *via* the Nrf2/HO-1 signaling pathway [47–49]. The Nrf2/HO-1 pathway proteins were next examined, and the results showed that NBP pretreatment activated Nrf2, HO-1 proteins in renal I/R brain tissues and OGD/R-BMVECs. This also confirmed that NBP pretreatment mediated antioxidant responses mainly through activation of the Nrf2/HO-1 pathway improving brain tissue damage.

How does NBP inhibit the inflammatory responthese in the brain due to renal I/R? Studies have shown that NF-κB is a major regulator of inflammation and immune homeostasis in the body [50,51]. Inhibition of the NOD2/MAPK/NF-κB signaling pathway is also thought to alleviate brain injury in I/R [52]. However, whether NBP inhibition of inflammatory response in renal I/R secondary brain injury is related to the NOD2/MAPK/NF-κB signaling pathway has not been reported. To investigate this question, MDP, a NOD2 agonist was used. The results suggested that NBP pretreatment inhibited the expression levels of NOD2/MAPK/NF-κB signaling pathway in both *in vivo* and *in vitro* models of I/R. Meanwhile, NOD2 activator MDP-mediated rescue experiments partially antagonized the cytoprotective function and inhibitory effect on inflammatory factors of NBP treatment in BMVECs, which suggests that the inhibition of the NOD2/MAPK/NF-κB pathway is critical for the brain protection exerted by NBP in rats with renal I/R rats.

Also, apoptosis and inflammation of BMVECs are believed to be pivotal in BBB injury. Findings from the *in vitro* OGD/R-BMVECs model suggest that NBP can reduce apoptosis, enhance cell viability, and improve tube-forming capacity. Importantly, this study also noted the role of paracellular tight junction proteins in maintaining BBB function [53]. Claudin-5 and ZO-1 maintain BBB integrity and reduce permeability *via* intercellular junctions [54]. The present investigation reveals that NBP pretreatment promotes the expression of Claudin-5 and ZO-1 in brain tissues and BMVECs. Notably, VEGF and vWF were also stimulated in brain tissues and BMVECs. VEGF is thought to play a dual role in brain injury. On the one hand, VEGF promotes BBB permeability and exacerbates neurological injury [55,56]. On the other hand, VEGF as a major angiogenic factor was shown to promote neurological repair by inducing neovascularization [57,58]. Similarly, vWF can modulate BBB flexibility and promote vascular remodeling during the recovery period [59]. Apparently, activation of VEGF and vWF by NBP appears to have a neuroprotective impact during the repair phase. These results suggest that NBP can alleviate secondary brain injury by protecting BBB integrity in early renal I/R and by promoting neovascularization and vascular remodeling during recovery. However, the molecular mechanisms need to be further investigated.

Of course, there are some limitations in this experiment, and the role of NBP in brain injury secondary to renal I/R needs to be validated in clinical samples in the future. In addition, how to prevent BBB disorder secondary to renal I/R at an early stage is also a focus of future research.

In conclusion, this study suggests that NBP inhibits brain injury following renal I/R through intricate pathological mechanisms, including reducing inflammation and oxidative stress levels, which are associated with Nrf2/HO-1 pathway activation, and NOD2/MAPK/NF-κB pathway inhibition. This study provides new scientific evidence for the clinical management of secondary renal I/R brain injury.

## Supplementary Material

Supplemental Material

## Data Availability

The data that support the findings of this study are available upon request.
