# Supplementary material for: Butylphthalide improves brain damage induced by renal ischemia-reperfusion injury rats through Nrf2/HO-1 and NOD2/MAPK/NF-κB pathways
Source: Ren Fail. 2023 Sep 21;45(2):2259234. doi: 10.1080/0886022X.2023.2259234 (PMC10515692; doi:10.1080/0886022X.2023.2259234)
Supplement: Supplemental Material [file IRNF_A_2259234_SM5580.pdf]

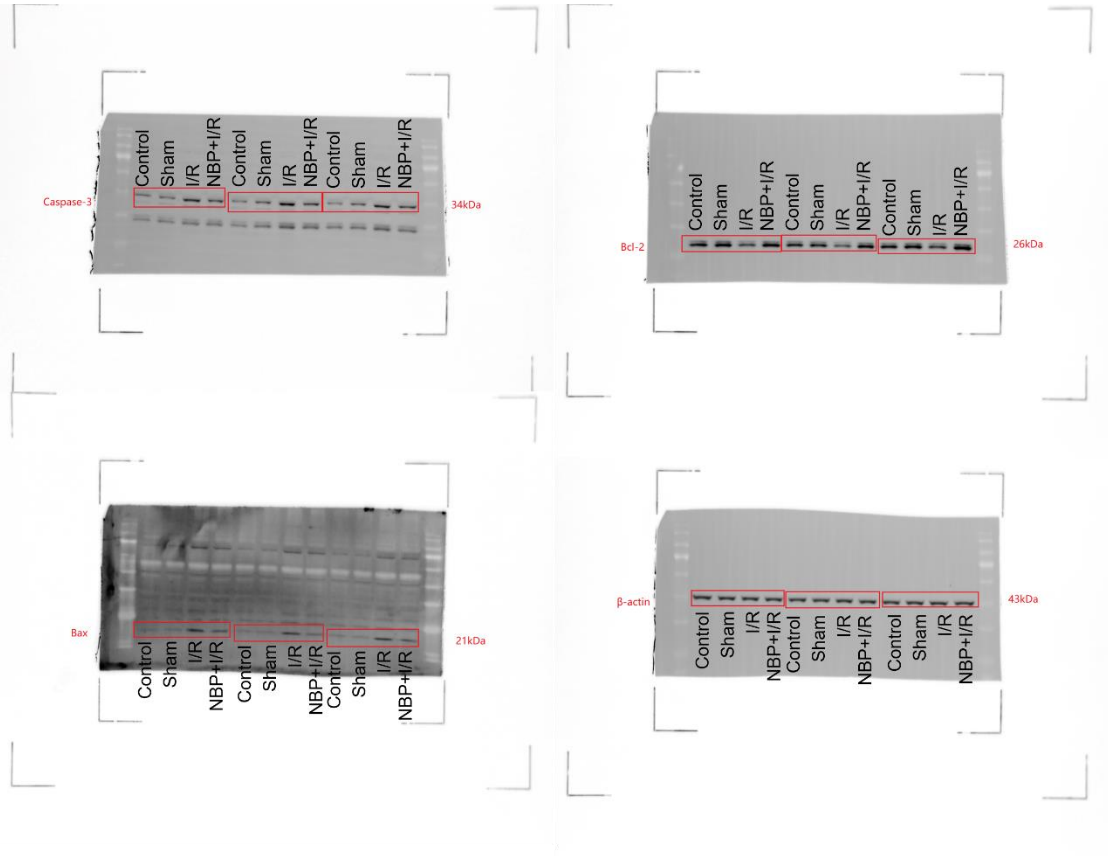

Supplementary Figure 1 The original Western blots of Caspase-3, Bcl-2, Bax, and β-actin in the cerebral cortex.

Information Classification: General

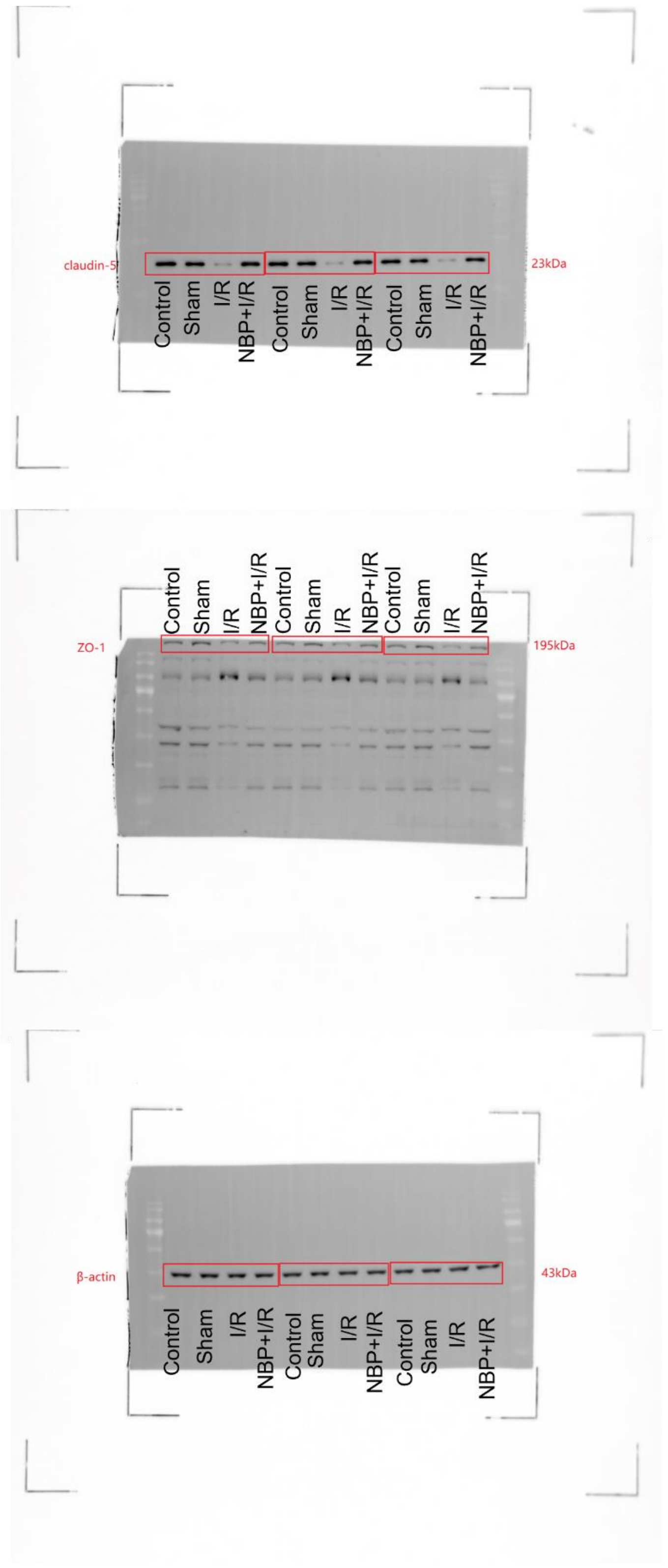

Supplementary Figure 2 The original Western blots of claudin-5, ZO-1, and β-actin in the brain of rats.

Information Classification: General

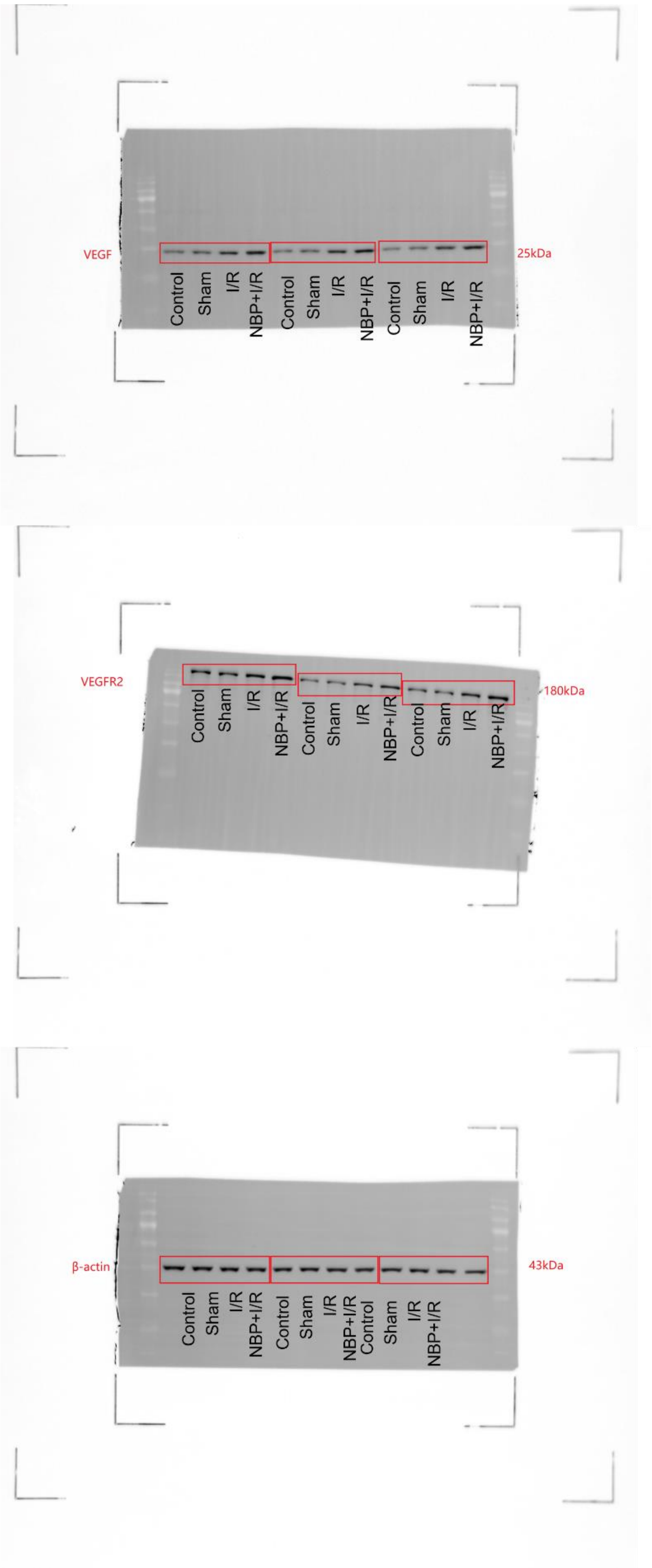

Supplementary Figure 3 The original Western blots of VEGF, VEGFR2, and β-actin in the brain of rats.

Information Classification: General

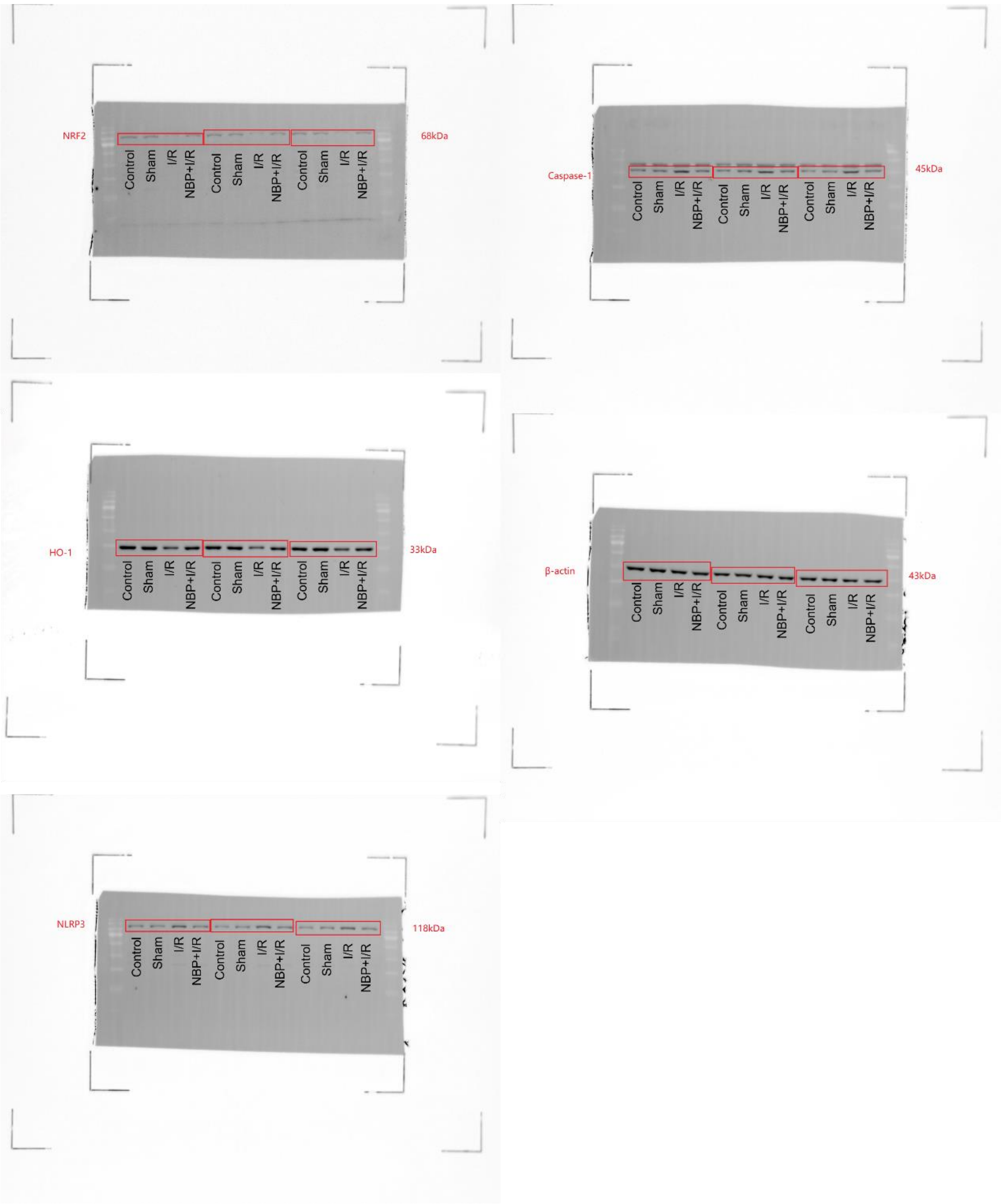

Supplementary Figure 4 The original Western blots of Nrf2, HO-1, NLRP3, Caspase-1, and β-actin in the brain of rats.

Information Classification: General

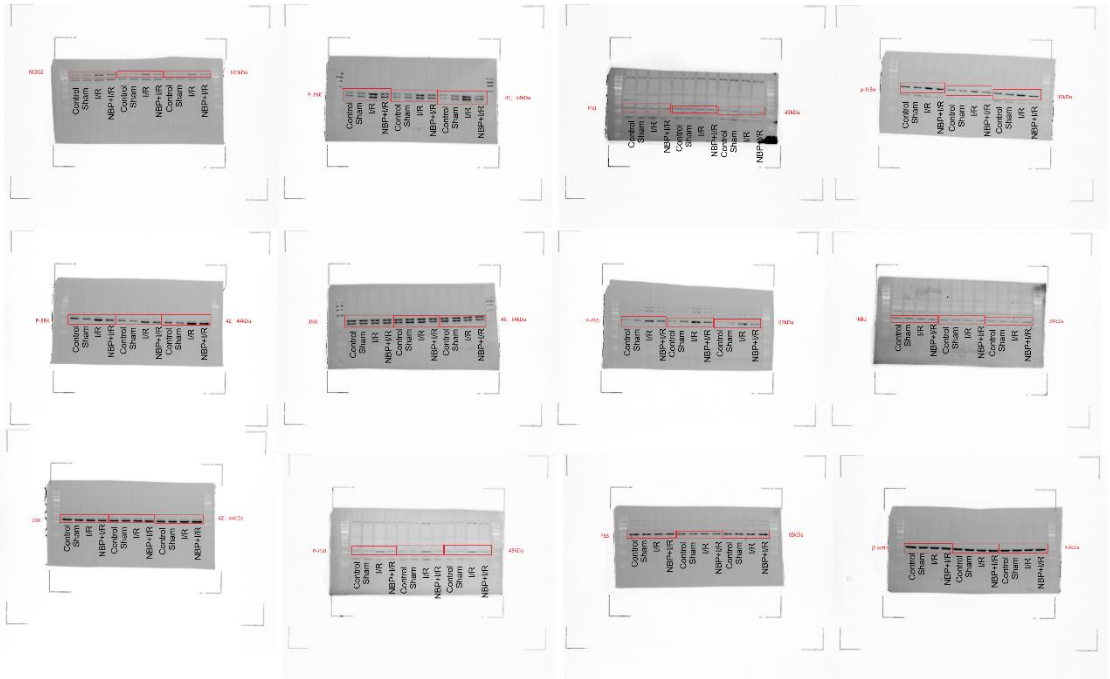

Supplementary Figure 5 The original Western blots of NOD2, p-ERK1/2, ERK1/2, p-JNK, JNK, p-p38 MAPK, p38 MAPK, p-NF-κB p65, NF-κB p65, p-IkBa, IkBa, and β-actin in the brain of rats.

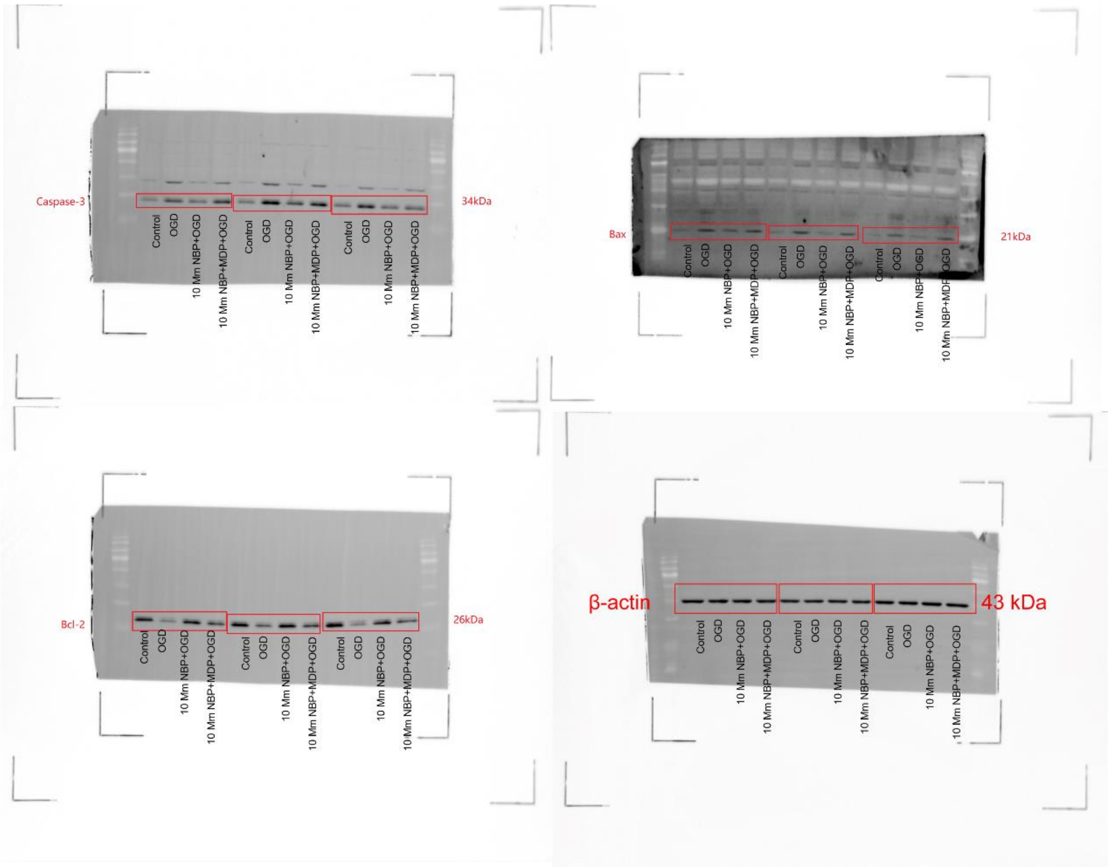

Supplementary Figure 6 The original Western blots of Caspase-3, Bcl-2, Bax, and β-actin in BMVECs.

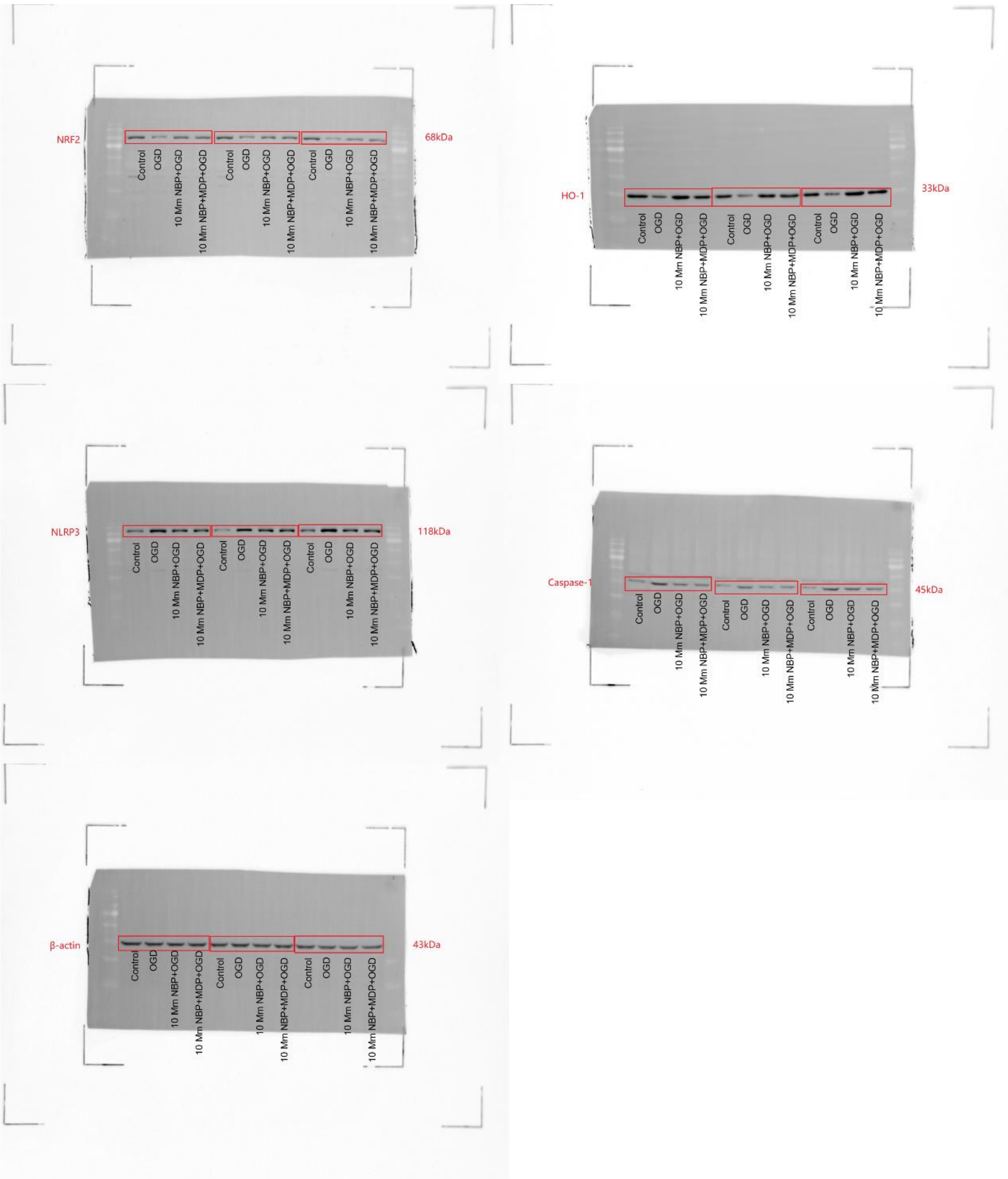

Supplementary Figure 7 The original Western blots of Nrf2, HO-1, NLRP3, Caspase-1, and β-actin in BMVECs.

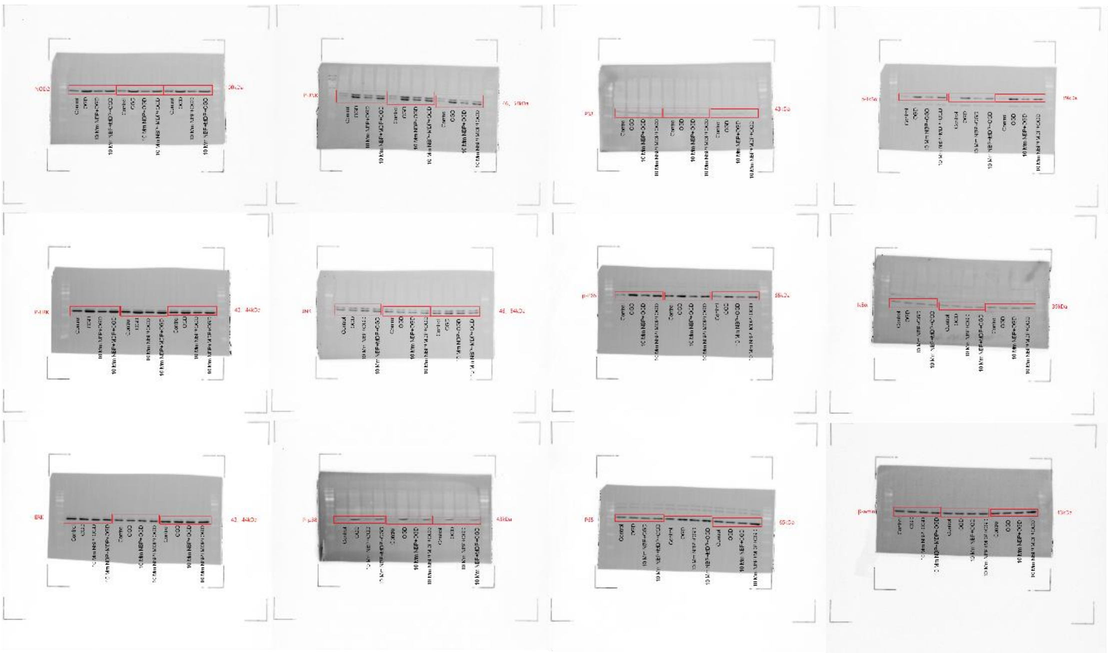

Supplementary Figure 8 The original Western blots of NOD2, p-ERK1/2, ERK1/2, p-JNK, JNK, p-p38 MAPK, p38 MAPK, p-NF-κB p65, NF-κB p65, p-IkBa, IkBa, and β-actin in BMVECs.

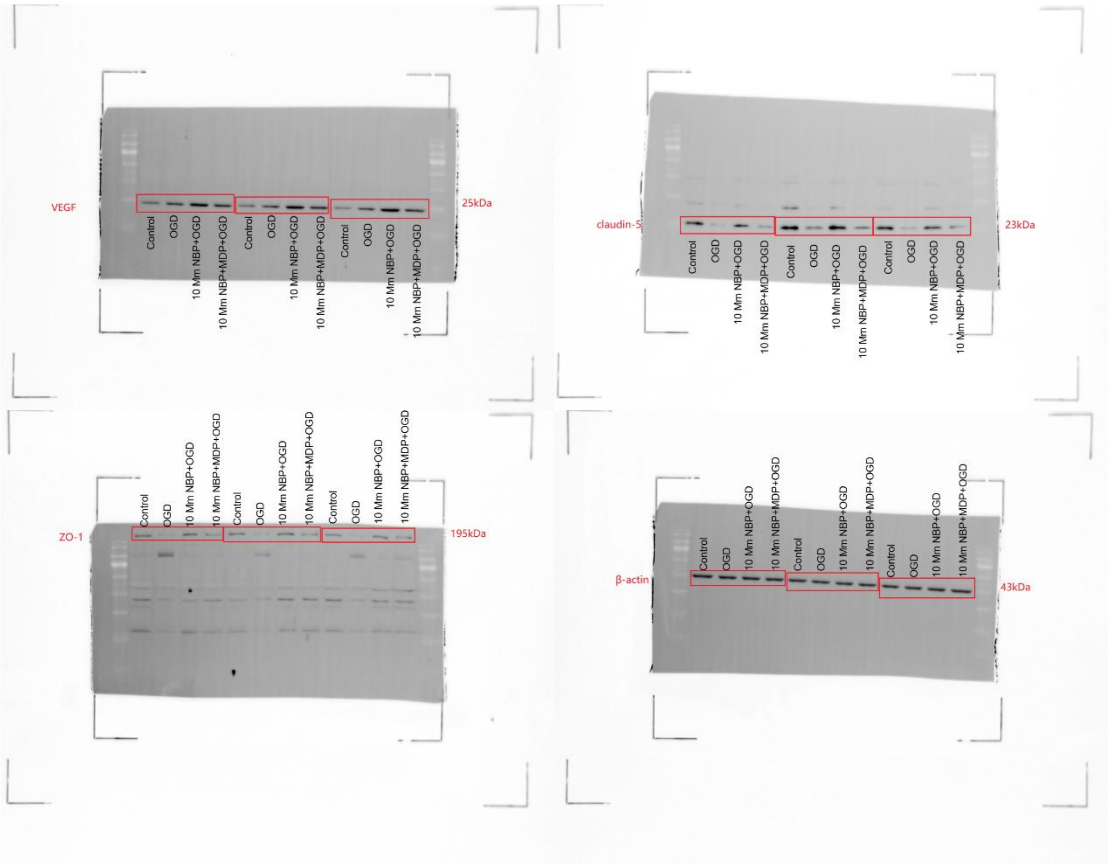

Supplementary Figure 9 The original Western blots of VEGF, Claudin-5, ZO-1, and β-actin in BMVECs.
